# Draft Genome Sequence of *Rhodococcus rhodochrous* Strain KG-21, a Soil Isolate from Oil Fields of Krishna-Godavari Basin, India

**DOI:** 10.1128/genomeA.01201-15

**Published:** 2015-10-15

**Authors:** Chhavi Dawar, Ramesh K. Aggarwal

**Affiliations:** Centre for Cellular and Molecular Biology (CSIR-CCMB), Tarnaka, Hyderabad, India

## Abstract

Here, we present the 6.1-Mb draft genome sequence of *Rhodococcus rhodochrous* strain KG-21, a soil isolate from the oil fields of Krishna-Godavari Basin in Andhra Pradesh, India. This genomic resource may help in the identification of the gene(s) involved in hydrocarbon degradation and their possible deployment for bioremediation.

## GENOME ANNOUNCEMENT

*Rhodococcus* includes a diverse group of bacteria within the wider nocardioform actinomycetes, which are commonly found in many environmental niches, from soils to seawater plants (1–3). *Rhodococcus* species are ideal candidates for enhancing the bioremediation of contaminated sites and also have been proven to be of immense use for a wide range of biotransformations, such as steroid modifications, enantioselective synthesis, and the production of amides from nitriles. Rhodococci can also catabolize short- and long-chain alkanes and aromatic (halogenated and nitro-substituted), heterocyclic, and polycyclic aromatic compounds (1–5). *Rhodococcus rhodochrous* strain KG-21, described here, was obtained from soil samples collected from a depth of 2.0 m from oil fields of the Krishna-Godavari (KG) Basin in Andhra Pradesh, India. The isolate identity was confirmed to the species level by 16S rRNA typing.

Genome sequencing for *R. rhodochrous* KG-21 was performed using the 454 (Roche FLX Titanium) pyrosequencing platform. A total of 73,640,254 bp were sequenced, spanning 183,571 reads. These reads were assembled using GS *De Novo* Assembler (version 2.8), generating a total of 236 contigs with an average size (*N*_50_) of 51 Kb and the longest contig size of 0.28 Mb. The draft genome sequence has a G+C content of 69.6%. Annotation of the draft genome was performed using the NCBI Prokaryotic Genome Annotation Pipeline (PGAP) and Rapid Annotations using Subsystem Technology (RAST) server (6). Pathway analysis studies were done using the KEGG Automatic Annotation Server (KAAS) (7), which provided functional annotation of the genes.

Genome annotation using PGAP revealed 5,577 genes, 5,222 coding sequences (CDS), 6 rRNAs, 52 tRNAs, and 3 noncoding RNAs (ncRNAs). RAST predicted 414 subsystem categories of metabolism, of which the subcategory of aromatic compound metabolism comprised 152 genes. Furthermore, several gene clusters (related to the catechol branch of the beta-ketoadipate pathway, biphenyl degradation, aromatic amine catabolism pathway, and specific denitrifying reductase) under the nitrogen metabolism category were uniquely annotated in KG-21, compared to its nearest neighbor *Rhodococcus jostii* RHA1 (RAST score, 516). KAAS pathway analysis functionally annotated several important microbial biotransformation genes in the new isolate KG-21, similar to earlier reports on gene cluster analysis for the genus *Rhodococcus* (8). Some of these included genes involved in the degradation of aromatic compounds, fatty acids, polycyclic aromatic hydrocarbons, chloroalkane/alkene, chlorocyclohexane/benzene, as well as, genes involved in specific chemical degradation pathways for limonene, pinene, geraniol, benzoate, toluene, xylene, styrene, atrazine, caprolactam, bisphenol, dioxin, and steroids.

Annotation of the draft genome sequence of KG-21 also revealed several genes that may help produce large amounts of lipids in the form of triacylglycerols (TAGs), due to the high activity of enzymes, like wax ester synthase/diacylglycerol acyltransferase, acetyl-coenzyme A (CoA) carboxylase, acyl carrier proteins, (1-acylglycerol-phosphate) acyltransferase, glycerol kinase, and glycerol-3-phosphate dehydrogenase, as reported earlier for *R. rhodochrous* strain ATCC 21198 (9). In addition, it also has genes for cytochrome P450 monooxygenase and several peroxidases, oxygenases, and lipases.

The availability of the *R. rhodochrous* KG-21 genome sequence will assist in understanding the potential metabolic capabilities of this organism and its application as a resource for biotechnological applications, like industrial wastewater management and oil spill degradation.

### Nucleotide sequence accession number.

This whole-genome shotgun project has been deposited in DDBJ/EMBL/GenBank under the accession no. AZYO00000000. The version described in this paper is the first version.
