# Correction for Dawar and Aggarwal, “Draft Genome Sequence of Rhodococcus rhodochrous Strain KG-21, a Soil Isolate from Oil Fields of Krishna-Godavari Basin, India”

**DOI:** 10.1128/MRA.01167-20

**Published:** 2020-10-29

**Authors:** Chhavi Dawar, Ramesh K. Aggarwal

**Affiliations:** a Centre for Cellular and Molecular Biology (CSIR-CCMB), Tarnaka, Hyderabad, India; b Academy of Scientific and Innovative Research (AcSIR), Ghaziabad, India

## AUTHOR CORRECTION

Volume 3, no. 5, e01201-15, 2015, https://doi.org/10.1128/genomeA.01201-15. Page 1: The author affiliations should appear as shown in this correction.

